# Effects of pyruvate administration on mRNA expression of inflammatory cytokines in adipose tissue and whole‐body glucose metabolism in male mice

**DOI:** 10.14814/phy2.70362

**Published:** 2025-08-09

**Authors:** Taichi Ando, Reo Takeda, Ryotaro Kano, Tatsuya Kusano, Yudai Nonaka, Yutaka Kano, Daisuke Hoshino

**Affiliations:** ^1^ Bioscience and Technology Program, Department of Engineering Science The University of Electro‐Communications Tokyo Japan; ^2^ Cellular and Molecular Biotechnology Research Institute National Institute of Advanced Industrial Science and Technology (AIST) Ibaraki Japan; ^3^ Research Fellowship for Young Scientists Japan Society for the Promotion of Science Tokyo Japan; ^4^ Institute of Liberal Arts and Science, Kakuma‐Machi Kanazawa University Kanazawa Japan

**Keywords:** IL‐6, lactate, obesity, white adipose tissue

## Abstract

Pyruvate administration leads to the accumulation of intracellular lactate in adipocytes and affects inflammatory cytokine production and whole‐body glucose metabolism. Therefore, the purpose of this study was to determine whether pyruvate administration improves the dysfunction of glucose metabolism induced by high‐fat diet (HFD) intake. In an acute experiment, intraperitoneal injection of male mice with sodium pyruvate (1 g/kg body weight) increased pyruvate and lactate concentrations in blood and epididymal white adipose tissue (eWAT). In a chronic experiment, male mice were divided into three groups: normal diet, HFD with saline administration (HFD + SAL), and HFD with sodium pyruvate administration (HFD + PYR); the HFD + PYR group was injected with pyruvate five times a week for 8 weeks. Insulin concentrations in the basal state and during an oral glucose tolerance test were significantly lower in the HFD + PYR group than in the HFD + SAL group. The mRNA expression of inflammatory cytokines (tumor necrosis factor‐alpha and interleukin 6) and a M2 macrophage polarity marker in eWAT was significantly higher in the HFD + PYR group than in the other groups. These results suggest that chronic pyruvate administration partially improves whole‐body glucose metabolic dysfunction in HFD‐fed mice, accompanied by increased mRNA expression of inflammatory cytokines and a M2 macrophage marker in the eWAT.

## 1 INTRODUCTION

Obesity caused by long‐term high‐fat diet (HFD) intake in rodents induces insulin resistance and impairs whole‐body glucose metabolism (Hossain et al., 2023; Nagy & Einwallner, 2018; Sharma et al., 2022). This dysfunction of systemic glucose metabolism is considered to be caused by the increased production of inflammatory cytokines, for example, tumor necrosis factor‐alpha (TNF‐α) and interleukin 6 (IL‐6), due to chronic inflammation in adipose tissue, especially visceral fat (Al‐Mansoori et al., 2022; Chawla et al., 2011; Hossain et al., 2023; Hotamisligil et al., 1993). This idea is strongly supported by studies using animal models deficient in these cytokines and their receptors (Cheung et al., 1998; Han et al., 2020; Kurauti et al., 2017). However, the relationship between inflammatory cytokines and glucose metabolic dysfunction has recently been questioned (Rosen & Kajimura, 2024). For example, systemic insulin resistance caused by HFD intake is preceded by the increased production of inflammatory cytokines in adipose tissue (Shimobayashi et al., 2018). Inflammatory cytokine signaling is rather necessary for the appropriate remodeling and expansion of adipose tissue (Asterholm et al., 2014). Furthermore, IL‐6 administration improves glucose and insulin homeostasis in obese mice (Peppler et al., 2019). In contrast, inhibition of an inflammatory cytokine signaling pathway in adipocytes impairs systemic glucose metabolism in mice (Zhu et al., 2020). These results suggest the possibility that inflammatory cytokines may have an important role in maintaining the health of adipose tissue and improving systemic glucose metabolism.

The production of inflammatory cytokines in adipocytes is regulated by various mechanisms (Ren et al., 2022). Among them, an increase in intracellular lactate accumulation triggers an increase in inflammatory cytokines in adipocytes. The adipocyte‐specific deletion of Slc16a1, encoding monocarboxylate transporter 1 (MCT1), which is responsible for lactate transport, upregulates IL‐6 mRNA expression in 3T3L1 adipocytes along with an increase in intracellular lactate accumulation due to the inability of cells to release lactate (Lin et al., 2022). In contrast, the adipocyte‐specific deletion of lactate dehydrogenase A, which converts pyruvate to lactate, lowers the percentage of inflammatory macrophages and decreases the production of inflammatory cytokines such as IL‐1β (Feng et al., 2022). Therefore, interventions that result in the accumulation of intracellular lactate in adipocytes may increase the production of inflammatory cytokines.

One potential intervention to increase lactate concentration in adipocytes in vivo is the administration of pyruvate. Adipocytes express MCT1, which has a high affinity for pyruvate (Bonen et al., 2006; Lin et al., 2022), and have a lactate‐to‐pyruvate concentration ratio of >10 and >100 at rest and during exercise, respectively, in vivo (Henderson et al., 2007; Parolin et al., 1999). No studies have examined whether pyruvate administration increases lactate concentration in adipocytes or adipose tissue, but the intraperitoneal (i.p.) injection of pyruvate to mice results in a twofold increase in blood lactate concentration (Soto et al., 2018), suggesting that pyruvate is converted to lactate elsewhere in the body. Adipose tissue is an active producer of lactate in humans (Digirolamo et al., 1992; Hellström et al., 1997; Jansson et al., 1990) and rats (Newby et al., 1989; Thacker et al., 1987). For example, 50%–65% of the glucose metabolized by adipocytes is converted to lactate (Digirolamo et al., 1992; Mårin et al., 1987; Thacker et al., 1987). These studies allow us to expect that administered pyruvate will be taken up by adipocytes and converted to lactate. Although there is a previous study using pyruvate administration with drinking water (Hasan et al., 2024), transient pyruvate administration, like an injection to increase blood lactate concentration, has not been examined. Therefore, we hypothesized that (1) acute pyruvate administration would increase lactate concentrations in adipose tissue, and (2) chronic pyruvate administration would alter the production of inflammatory cytokines and ameliorate whole‐body glucose metabolic dysfunction caused by HFD intake.

Furthermore, the conversion of pyruvate to lactate is accompanied by the production of nicotinamide adenine dinucleotide (NAD^+^). Since reduced NAD^+^ is associated with metabolic dysfunction (Imai & Guarente, 2014), increasing NAD^+^ through pyruvate administration may also contribute to improving systemic glucose metabolism. In fact, it has been reported that restoring NAD^+^ levels in HFD‐induced obese mice improves glucose intolerance (Yoshino et al., 2011). Although the previous study showed that chronic pyruvate administration ameliorated HFD‐induced abnormalities in glucose metabolism (Hasan et al., 2024), pyruvate and lactate concentrations after pyruvate administration were not examined. Therefore, we examined the effects of acute pyruvate injection on lactate and pyruvate concentrations in various tissues of mice and also assessed the effects of chronic pyruvate injection on inflammatory cytokine mRNA expression in adipose tissue and systemic glucose metabolism in HFD‐fed mice.

## 2 MATERIALS AND METHODS

### 2.1 Animals

Six‐week‐old male C57BL/6J mice (*n* = 40) were obtained from SLC Japan (Shizuoka, Japan). All mice were housed in a temperature‐controlled room (21–24°C) with a 12‐h light/dark cycle and were allowed food and water ad libitum (Rodent Lab Diet EQ, 5 L37; PMI Nutrition International, St. Louis, MO). All experiments in this study were approved by the Institutional Animal Care and Use Committee of the University of Electro‐Communications (no. A33).

### 2.2 Experimental design

#### 2.2.1 Experiment 1: Single administration of pyruvate

Sodium pyruvate (P2256; Sigma Aldrich, St. Louis, MO) solution was injected i.p. into 10‐week‐old male C57BL/6J mice (1.0 g/kg body weight [BW]) in the awake state, referring to a previous study (Soto et al., 2018). Lactate concentrations were measured in blood taken from the tail vein using Lactate Pro 2 (Arkray, Inc., Kyoto, Japan) before and at 10, 30, 60, and 120 min after pyruvate injection. Male C57BL/6J mice (10–13 weeks old) were randomly divided into a saline (SAL: *n* = 8) or pyruvate (PYR: *n* = 8) group; the SAL group was injected i.p. with saline, while the PYR group was injected i.p. with 1.0 g/kg BW of sodium pyruvate solution. The SAL and PYR mice were fasted after injection. Tissues were harvested at 10 min after injection.

#### 2.2.2 Experiment 2: Chronic administration of pyruvate

Eight‐week‐old male C57BL/6J mice were randomly divided into three groups: normal diet (ND: *n* = 7), HFD and saline injection (HFD + SAL: *n* = 7), or HFD and pyruvate injection (HFD + PYR: *n* = 6). The ND contained 10% fat (D12492; Research Diets, Inc., New Brunswick, NJ) and the HFD contained 60% fat (D12450J; Research Diets, Inc.). The HFD + SAL mice were injected i.p. with saline, and the HFD + PYR mice were injected i.p. with 1.0 g/kg BW of sodium pyruvate solution. The mice were injected once a day between 15:00 and 17:00, 5 days a week for 8 weeks.

### 2.3 Oral glucose tolerance test (OGTT)

At 7 weeks after the intervention in Experiment 2, oral glucose (1.0 g/kg BW) was administered after fasting for 12 h. Tail blood samples were collected before and at 10, 30, 60, and 120 min after glucose administration. After the samples were centrifuged at 10,000 × *g*, the plasma was collected and stored at −80°C.

### 2.4 Metabolic assessments

At 7 weeks after the intervention in Experiment 2, VO_2_ and VCO_2_ were measured in mice (*n* = 4 each group) from the HFD + SAL and HFD + PYR groups using a metabolic gas analysis system (ARCO‐2000; Arco Systems, Inc., Chiba, Japan). All mice were acclimated for 2 days in an individual chamber (W120 × H150 × D240 mm) with free access to food and water. Data were acquired every 10 min and averaged per hour. Locomotion was measured using an Actracer 20,000 SN (Arco Systems, Inc.).

### 2.5 Tissue sample collection

In Experiment 1, blood, soleus muscle, plantaris muscle, liver, and epididymal white adipose tissue (eWAT) were harvested under isoflurane anesthesia at 10 min after pyruvate administration. In Experiment 2, after 8 weeks of intervention, blood, soleus muscle, plantaris muscle, gastrocnemius muscle, eWAT, inguinal subcutaneous white adipose tissue, and brown adipose tissue were collected after fasting for 3 h. Tissue samples were frozen in liquid nitrogen and stored at −80°C. Blood samples were centrifuged at 10,000 × *g* for 10 min, and the plasma was collected and stored at −80°C.

### 2.6 Blood glucose and insulin concentrations

Plasma glucose and insulin levels were measured using a Glucose C2 Test Wako Kit (439‐90901; Fujifilm Wako Pure Chemical, Osaka, Japan) and a Mouse Insulin ELISA Kit (10‐1247‐01; Mercodia AB, Uppsala, Sweden), respectively. The Homeostatic Model Assessment for Insulin Resistance (HOMA‐IR) was performed using plasma glucose and insulin concentrations at rest. The areas under the curve (AUCs) were calculated with baseline as a value of 0 min during the OGTT.

### 2.7 Blood and tissue pyruvate concentrations

Blood and tissue pyruvate concentrations were measured using a Pyruvate Assay Kit (EPRK‐100; BioAssay Systems, Hayward, CA). Tissues were homogenized in a bead crusher (μT‐01; TITEC, Saitama, Japan) with a 20‐fold volume of 0.3 M perchloric acid, and after centrifugation (3000 × *g* for 10 min at 4°C), the samples were neutralized using 1.0 M NaOH. Pyruvate concentrations were measured in blood and muscle homogenates using a 96‐well plate reader (Thermo Fisher Scientific, Waltham, MA).

### 2.8 Tissue lactate concentrations

Lactate concentrations were measured as described previously (Hoshino et al., 2015). Briefly, 10 μL neutralized sample, as used for the pyruvate measurements, was mixed with 230 μL lactate assay solution (0.4 M hydrazine, 0.5 M glycine, 0.4 mM NAD+, and 1000 U L‐lactate dehydrogenase). After incubation for 30 min at room temperature, absorbance at 340 nm was measured using a 96‐well plate reader (Thermo Fisher Scientific).

### 2.9 Quantitative PCR

Adipose tissue samples were homogenized in TRIzol reagent (15596026; Thermo Fisher Scientific). After homogenization, total RNA was isolated using a NucleoSpin RNA Kit (740955; Takara Bio, Shiga, Japan), and RNA concentration was quantified using a NanoDrop Lite (Thermo Fisher Scientific). Reverse transcription was performed using a High‐Capacity RNA‐to‐cDNA Kit (4387406; Thermo Fisher Scientific). Real‐time PCR was performed using SYBR Green (A25780; Thermo Fisher Scientific) on a StepOne System (Thermo Fisher Scientific). 18S ribosomal RNA was used as a control housekeeping gene. The primers used are shown in Table 1.

**TABLE 1:** Real‐time PCR primer sequences.

Gene	Forward primer (5′‐3′)	Reverse primer (5′‐3′)
18S ribosomal RNA	TTC TGG CCA ACG GTC TAG ACA AC	CCA GTG GTC TTG GTG TGC TGA
TNFα	GCC TCT TCT CAT TCC TGC TTG TG	TGA TGA GAG GGA GGC CAT TTG
MCP1	CTC ACC TGC TGC TAC TCA TTC	ACT ACA GCT TCT TTG GGA CAC
IL6	CCA GAG ATA CAA AGA AAT GAT GG	ACT CCA GAA GAC CAG AGG AAA T
CD68	CTT CCC ACA GGC AGC ACA G	AAT GAT GAG AGG CAG CAA GAG G
CXCL1	GCT GGC TTC TGA CAA CAC TAT	CAA GCA GAA CTG AAC TAC CAT
Nos2	CCA AGC CCT CAC CTA CTT CC	CTC TGA GGG CTG ACA CAA GG
Arg1	CTC CAA GCC AAA GTC CTT AGA G	AGG AGC TGT CAT TAG GGA CAT C
PPARγ	ATC TCT GTT TTA TGC TGT TAT GG	GCT CTT GTG AAT GGA ATG TCT T
C/EBPα	AGG TGC TGG AGT TGA CCA GT	CAG CCT AGA GAT CCA GCG AC
C/EBPβ	AAG CTG AGC GAC GAG TAC AAG A	GTC AGC TCC AGC ACC TTG TG
C/EBPδ	TCC ACG ACT CTG CCA TGT A	GCG GCC ATG GAG TCA ATG

Abbreviations: Arg1, arginase 1; CD68, cluster of differentiation 68; CEBP/α, β, δ, CCAAT/enhancer binding protein alpha, beta, gamma; CXCL, C‐X‐C motif chemokine ligand; IL6, interleukin‐6; MCP1, monocyte chemoattractant protein‐1; Nos2, nitric oxide synthase 2; PPARγ, peroxisome proliferator‐activated receptor gamma; TNFα, tumor necrosis factor‐alpha.

### 2.10 Western blot analysis

Adipose tissue samples were lysed in RIPA lysis buffer (20‐188; Millipore, Burlington, MA), as described previously (Amano et al., 2020; Takeda et al., 2023), containing protease inhibitor cocktail (11836170001; Sigma‐Aldrich) and phosphatase inhibitor (04906837001; Roche, Basel, Switzerland). Total cell lysate protein concentrations were quantified using a BCA Protein Assay Kit (23227; Thermo Fisher Scientific). Equal amounts of protein (10 μg) were loaded onto TGX gels (Bio‐Rad, Hercules, CA) and separated. Proteins were then transferred to polyvinylidene fluoride membranes and blocked with Bullet Blocking One (13779‐01; Nacalai Tesque, Kyoto, Japan). The membranes were incubated with primary antibodies (Table 2) overnight at 4°C. After incubation, the membranes were incubated with secondary antibodies (Table 2) for 1 h at room temperature. Bands were visualized with Chemi‐Lumi One (07880; Nacalai Tesque) or Chemi‐Lumi One Super (02230; Nacalai Tesque) and quantified with a ChemiDoc Imaging System (Bio‐Rad). Coomassie brilliant blue staining was performed to ensure equal protein loading.

**TABLE 2:** Primary and secondary antibodies.

Antibodeies	Company	Catalog number
GLUT4	Abcam	ab33780
Phospho‐AS160 (Thr642)	Cell Signaling Technology	4288
AS160	Cell Signaling Technology	2670
Phospho‐Akt (Ser473)	Cell Signaling Technology	4060
Akt	Cell Signaling Technology	4691
Anti‐rabbit IgG	Cell Signaling Technology	7074

### 2.11 Statistical analysis

All data are expressed as the mean ± standard deviation. Two‐way analysis of variance and post hoc analysis (Tukey) were carried out for time‐series data of glucose and insulin concentrations during the OGTT and for body weight and food intake over time during the intervention period (intervention × time). For other data, one‐way analysis of variance and post hoc analysis (Dunnett or Tukey) were used for time changes in one group and for comparisons among the three groups, and an unpaired *t*‐test was used for comparisons among two groups. Pearson's correlation coefficient was used to examine correlations. Statistical significance was set at *p* < 0.05. All statistical procedures were performed using Prism ver. 9 (GraphPad Software, San Diego, CA).

## 3 RESULTS

### 3.1 Blood and tissue pyruvate and lactate concentrations

**FIGURE 1:**
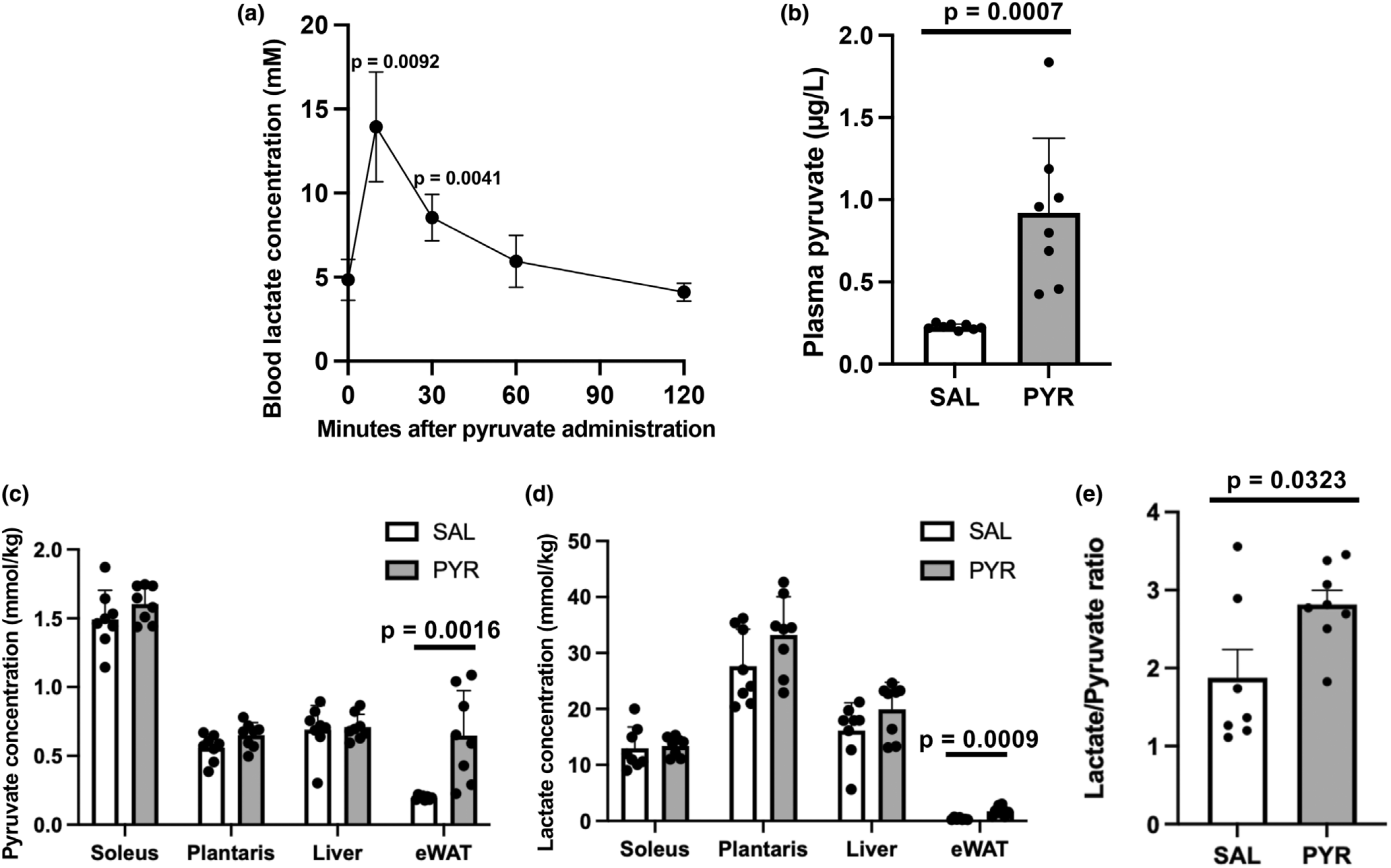
Lactate and pyruvate concentrations in plasma and tissues after pyruvate intraperitoneal (i.p.) injection to mice. (a) Blood lactate concentrations after 1 g/kg pyruvate i.p. injection. (b) Plasma pyruvate concentrations at 10 min after injection. (c) Pyruvate and (d) lactate concentrations in the soleus muscle, plantaris muscle, liver, and epididymis adipose tissue (eWAT) at 10 min after injection. (e) Lactate/pyruvate ratio in eWAT at 10 min after injection. Data are shown as the mean ± standard deviation. *n* = 6 (a), *n* = 7 each group (b–e). PYR, pyruvate; SAL, saline.

Lactate and pyruvate concentrations in blood and tissues were evaluated after pyruvate i.p. injection. Blood lactate concentrations were significantly higher at 10 and 30 min after pyruvate injection than before injection (10 min, +189%, *p* = 0.0092; 30 min, +76.6%, *p* = 0.0041; Figure 1a). Blood pyruvate concentrations were significantly higher in the PYR group than in the SAL group at 10 min after pyruvate injection (+305%, *p* = 0.0007; Figure 1b). While there were no significant differences in pyruvate and lactate concentrations in the soleus muscle, plantaris muscle, and liver at 10 min after pyruvate injection between the two groups, pyruvate and lactate concentrations in eWAT were significantly higher in the PYR group than in the SAL group (pyruvate, +229%, *p* = 0.0016; Figure 1c; lactate, +371%, *p* = 0.0009; Figure 1d). The lactate/pyruvate ratio in eWAT was also significantly higher in the PYR group than in the SAL group (+50%, *p* = 0.0323; Figure 1e).

### 3.2 Body weight, food intake, and gas assessment

**FIGURE 2:**
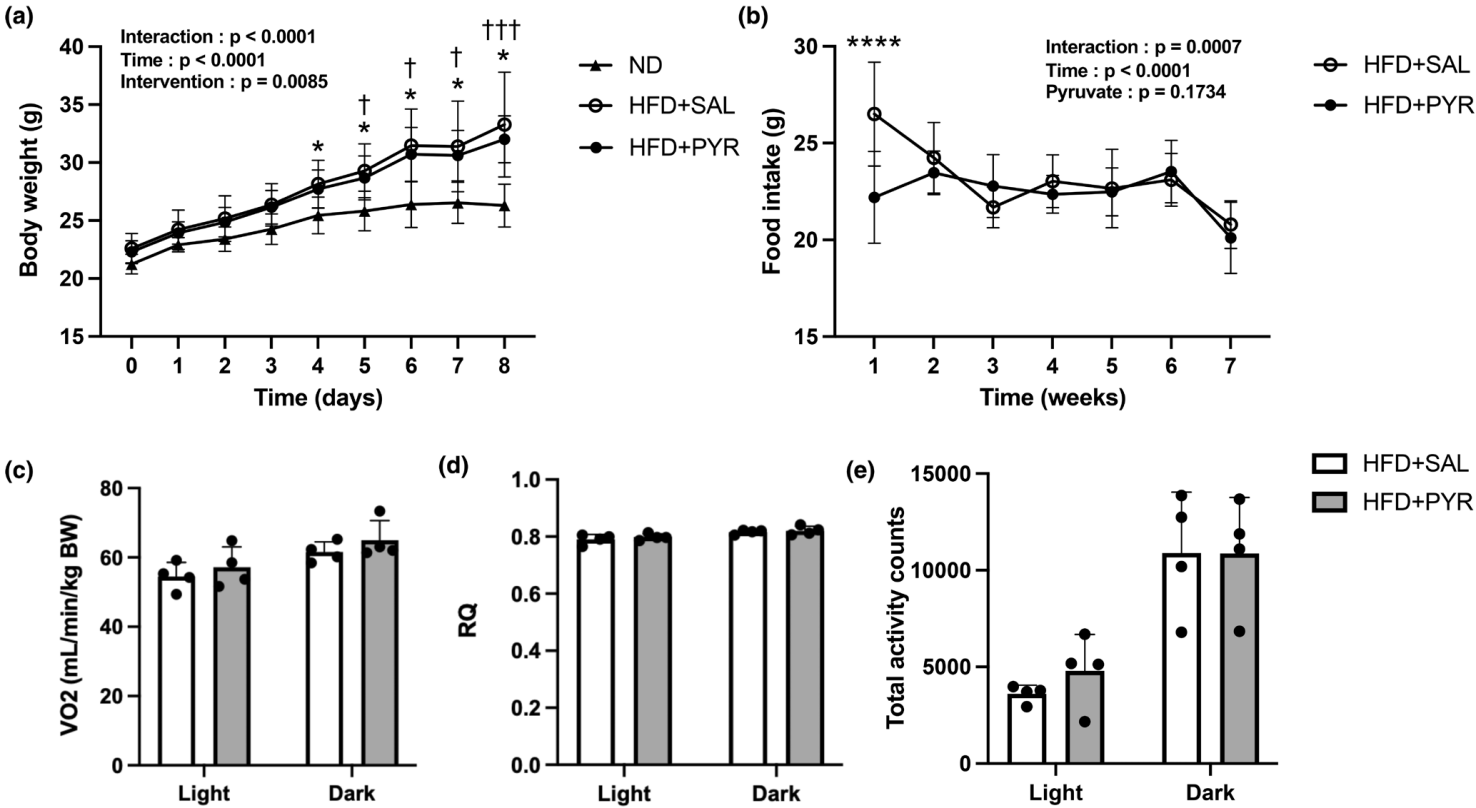
Metabolic assessments. (a) Body weight and (b) food intake during the 8‐week intervention period. (c) VO_2_, (d) respiratory quotient (RQ), and (e) amount of total activity counts in the light and dark periods. Data are shown as the mean ± standard deviation. *p < 0.05, ****p < 0.0001 HFD + SAL vs. ND (a), vs. HFD + PYR (b). ^†^ *p* < 0.05, ^†††^ *p* = 0.0008 HFD + PYR vs. ND. ND and HFD + SAL: *N* = 7, HFD + PYR: *N* = 6 (A, B), *n* = 4 each group (c–e). BW, body weight; HFD, high‐fat diet; ND, normal diet.

During the intervention period, the body weight of the HFD + SAL group was significantly higher than that of the ND group from the 4th week onward (+26.6%, *p* < 0.05, at the 8th week; Figure 2a). The body weight of the HFD + PYR group was significantly higher than that of the ND group from the 5th week onward (+21.7%, *p* < 0.001, at the 8th week; Figure 2a), but it was not significantly different between the HFD + SAL and HFD + PYR groups. Food intake was significantly lower in the HFD + PYR group than in the HFD + SAL group at the 1st week (−19.4%, *p* < 0.0001; Figure 2b) but was not significantly different after the 2nd week. VO_2_, respiratory quotient, and locomotive activity were not significantly different between the HFD + SAL and HFD + PYR groups in both the light and dark periods (Figure 2c–e).

### 3.3 OGTT

**FIGURE 3:**
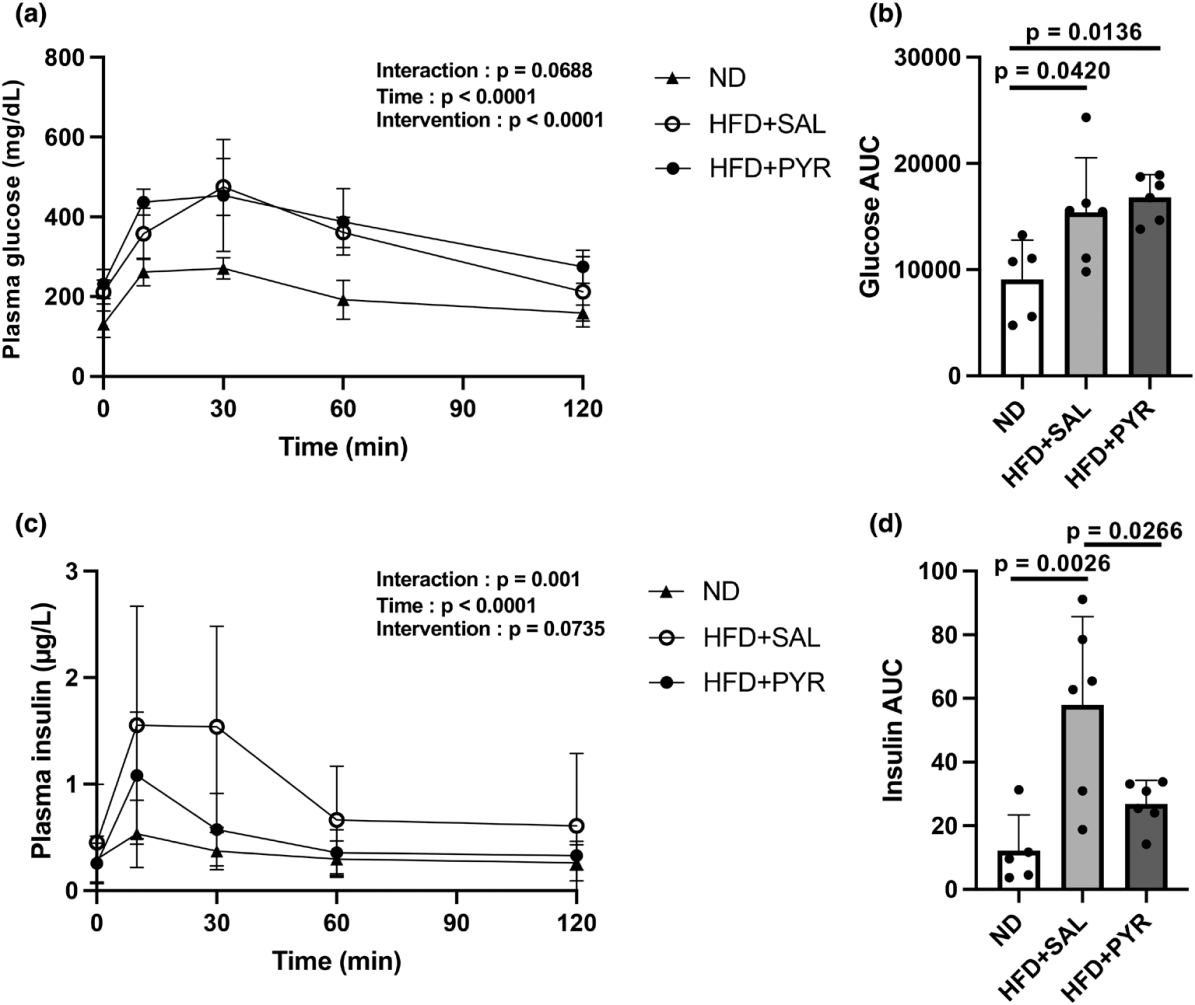
Plasma glucose and insulin concentrations during an oral glucose tolerance test. (a) Plasma glucose and (c) insulin concentrations after oral glucose administration. Areas under the curve (AUCs) for (b) plasma glucose and (d) plasma insulin throughout the 120‐min period after oral glucose administration. Data are shown as the mean ± standard deviation. ND: *N* = 5, HFD + SAL and HFD + PYR: *N* = 6.

An OGTT was performed to evaluate whole‐body glucose metabolism. There was no significant difference in blood glucose concentrations at any time point during the OGTT among the three groups (Figure 3a). While the AUC of glucose was significantly higher in the HFD + SAL and HFD + PYR groups than in the ND group (HFD + SAL, +69.7%, *p* = 0.042; HFD + PYR, +84.9%, *p* = 0.0136; Figure 3b), it was not significantly different between the HFD + SAL and HFD + PYR groups. Blood insulin concentrations over time during the OGTT showed a significant interaction between the three groups (*p* = 0.001; Figure 3c), but there was no significant difference at any time point between the groups. The AUC of insulin was significantly higher in the HFD + SAL group than in the ND (+375%, *p* = 0.0026; Figure 3d) and HFD + PYR (+115%, *p* = 0.0266; Figure 3d) groups. There was no significant difference in the AUC of insulin between the ND and HFD + PYR groups.

### 3.4 Tissue weight and plasma analysis

Soleus muscle, eWAT, inguinal subcutaneous white adipose tissue, and brown adipose tissue weights were significantly higher in the HFD + SAL group than in the ND group (soleus muscle, +20.3%, *p* < 0.05; eWAT, +200%, *p* < 0.01; inguinal subcutaneous white adipose tissue, +164%, *p* < 0.001; brown adipose tissue, +80.2%, *p* < 0.01; Table 3). In the basal state, blood insulin levels were significantly higher in the HFD + SAL group (+128%, *p* < 0.0001) and HFD + PYR (+69.4%, *p* < 0.05; Table 3) groups than in the ND group but were significantly lower in the HFD + PYR group than in the HFD + SAL group (−25.8%, *p* < 0.05; Table 3). The HOMA‐IR was also significantly higher in the HFD + SAL group than in the ND group (+136%, *p* < 0.001; Table 3) but was significantly lower in the HFD + PYR group than in the HFD + SAL group (−36.9%, *p* < 0.05; Table 3).

**TABLE 3:** Tissue weights and plasma glucose and insulin concentrations at rest.

	ND	HFD + SAL	HFD + PYR
SOL weight (mg)	9.1 ± 1.1	11.0 ± 1.3*	10.2 ± 1.5
PLA weight (mg)	15.3 ± 3.3	18.0 ± 1.9	18.3 ± 2.1
GAS weight (mg)	139.7 ± 13.3	150.9 ± 14.5	140.0 ± 13.3
eWAT weight (mg)	356.0 ± 90.0	1423.1 ± 841.7**	881.0 ± 280.3
iWAT weight (mg)	209.1 ± 73.8	761.3 ± 319.8***	517.7 ± 175.2
BAT weight (mg)	70.9 ± 10.9	127.7 ± 43.6**	102.2 ± 22.4
Plasma glucose (mg/dL)	280.4 ± 58.7	310 ± 22.5	265 ± 19.5
Plasma insulin (μg/L)	0.196 ± 0.053	0.448 ± 0.105****	0.333 ± 0.070* ^,^ ^†^
HOMA‐IR	0.146 ± 0.063	0.344 ± 0.086***	0.217 ± 0.048^†^

*Note*: Data are shown as the mean ± SD. The homeostatic model assessment for insulin resistance (HOMA‐IR) was calculated by plasma glucose and plasma insulin.

Abbreviations: BAT, brown adipose tissue; eWAT, epididymal white adipose tissue; GAS, gastrocnemius muscle; iWAT, inguinal subcutaneous white adipose tissue; PLA, plantaris muscle; SOL, soleus muscle.

* *p* < 0.05.

** *p* < 0.01.

*** *p* < 0.001.

**** *p* < 0.0001 vs. ND.

^†^ *p* < 0.05 vs. HFD + SAL.

### 3.5 mRNA expression of inflammatory cytokines and macrophage polarity markers in eWAT

**FIGURE 4:**
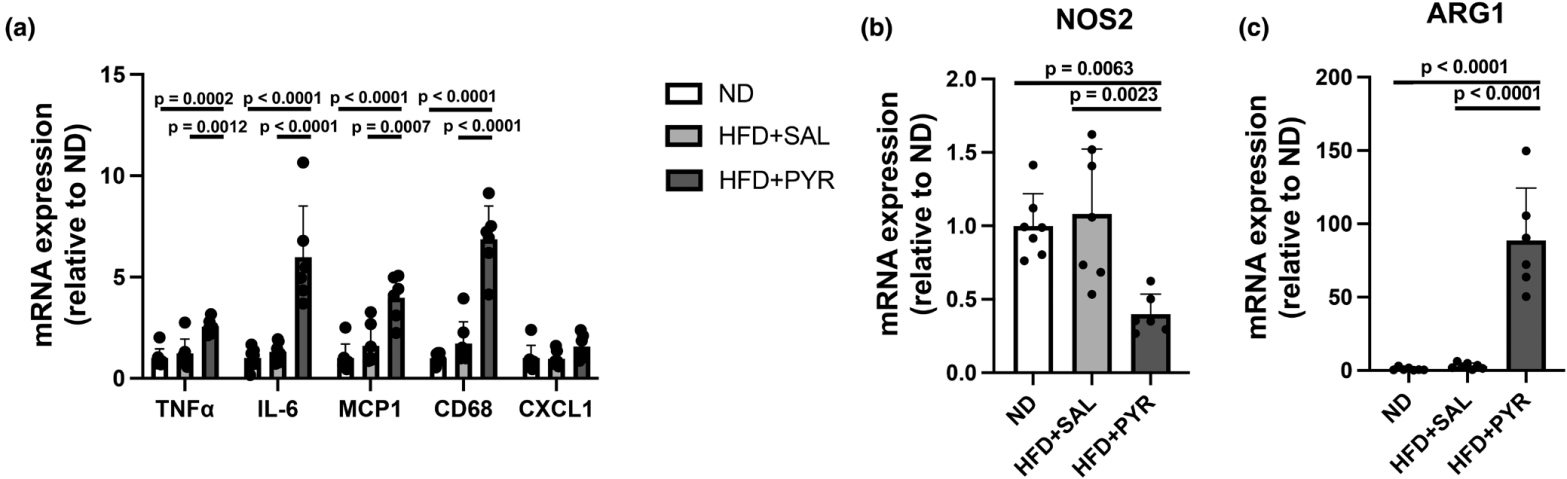
Effects of chronic pyruvate administration on the mRNA expression of inflammation and macrophage polarity markers in epididymal white adipose tissue. (a) Inflammatory cytokines. (b, c) Macrophage polarity markers. Data are shown as the mean ± standard deviation. ND and HFD + SAL: *N* = 7, HFD + PYR: *N* = 6.

The gene expression of inflammatory cytokines (TNF‐α, IL‐6, monocyte chemoattractant protein 1 [MCP1], cluster of differentiation 68 [CD68], and C‐X‐C motif chemokine ligand 1 [CXCL1]) and macrophage polarity markers (nitric oxide synthase 2 [NOS2] and arginase 1 [ARG1]) was measured in eWAT. TNFα, IL‐6, MCP1, and CD68 mRNA expression was significantly higher in the HFD + PYR group than in the ND (TNF‐α, +156%, *p* = 0.0002; IL‐6, +499%, *p* < 0.0001; MCP1, +298%, *p* < 0.0001; CD68, +586%, *p* < 0.0001; Figure 4a) and HFD + SAL (TNF‐α, +108%, *p* = 0.0012; IL‐6, +356%, *p* < 0.0001; MCP1, +148%, *p* = 0.0007; CD68, +301%, *p* < 0.0001; Figure 4a) groups. NOS2 gene expression was significantly lower in the HFD + PYR group than in the ND (−60.1%, *p* = 0.0063; Figure 4b) and HFD + SAL (−63.1%, *p* = 0.0023; Figure 4b) groups. ARG1 gene expression was significantly higher in the HFD + PYR group than in the ND (+8770%, *p* < 0.0001; Figure 4c) and HFD + SAL (+2867%, *p* < 0.0001; Figure 4c) groups. There was no significant difference in CXCL1 mRNA expression between the three groups.

### 3.6 mRNA expression of adipocyte differentiation markers in eWAT

**FIGURE 5:**
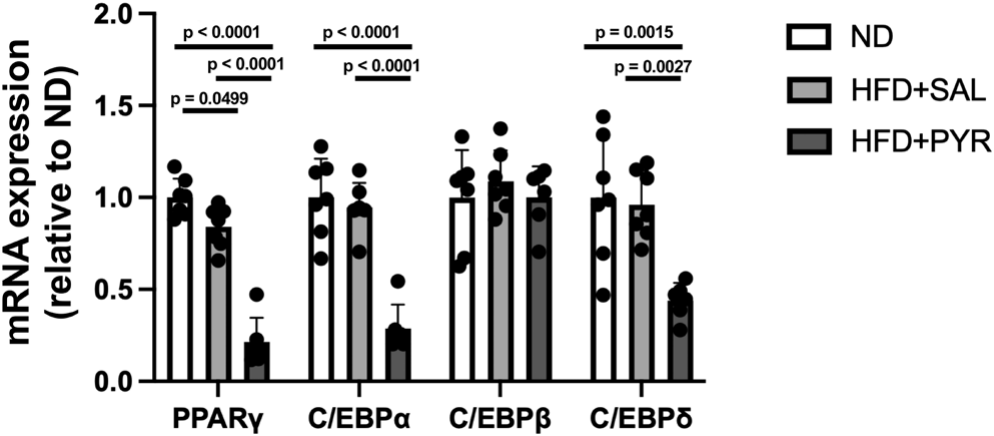
Effects of chronic pyruvate administration on the mRNA expression of adipocyte differentiation markers in epididymal white adipose tissue. Data are shown as the mean ± standard deviation. ND and HFD + SAL: *N* = 7, HFD + PYR: *N* = 6.

To evaluate the effect of the intervention on adipocyte differentiation, the gene expression of differentiation markers (peroxisome proliferator‐activated receptor γ [PPARγ] and CCAAT/enhancer binding protein [C/EBP] α, β, and δ) was measured in eWAT. PPARγ, C/EBPβ, and C/EBPδ mRNA expression was significantly lower in the HFD + PYR group than in the ND (PPARγ, −78.7%, *p* < 0.001; C/EBPα, −71.2%, *p* < 0.0001; C/EBPδ, −56%, *p* = 0.0015; Figure 5) and HFD + SAL (PPARγ, −74.6%, *p* < 0.0001; C/EBPα, −69.6%, *p* < 0.0001; C/EBPδ, −54.3%, *p* = 0.0027; Figure 5) groups. There was no significant difference in C/EBPβ mRNA expression between the three groups.

### 3.7 Relationship between IL‐6 mRNA expression in eWAT and plasma glucose and insulin concentrations

**FIGURE 6:**
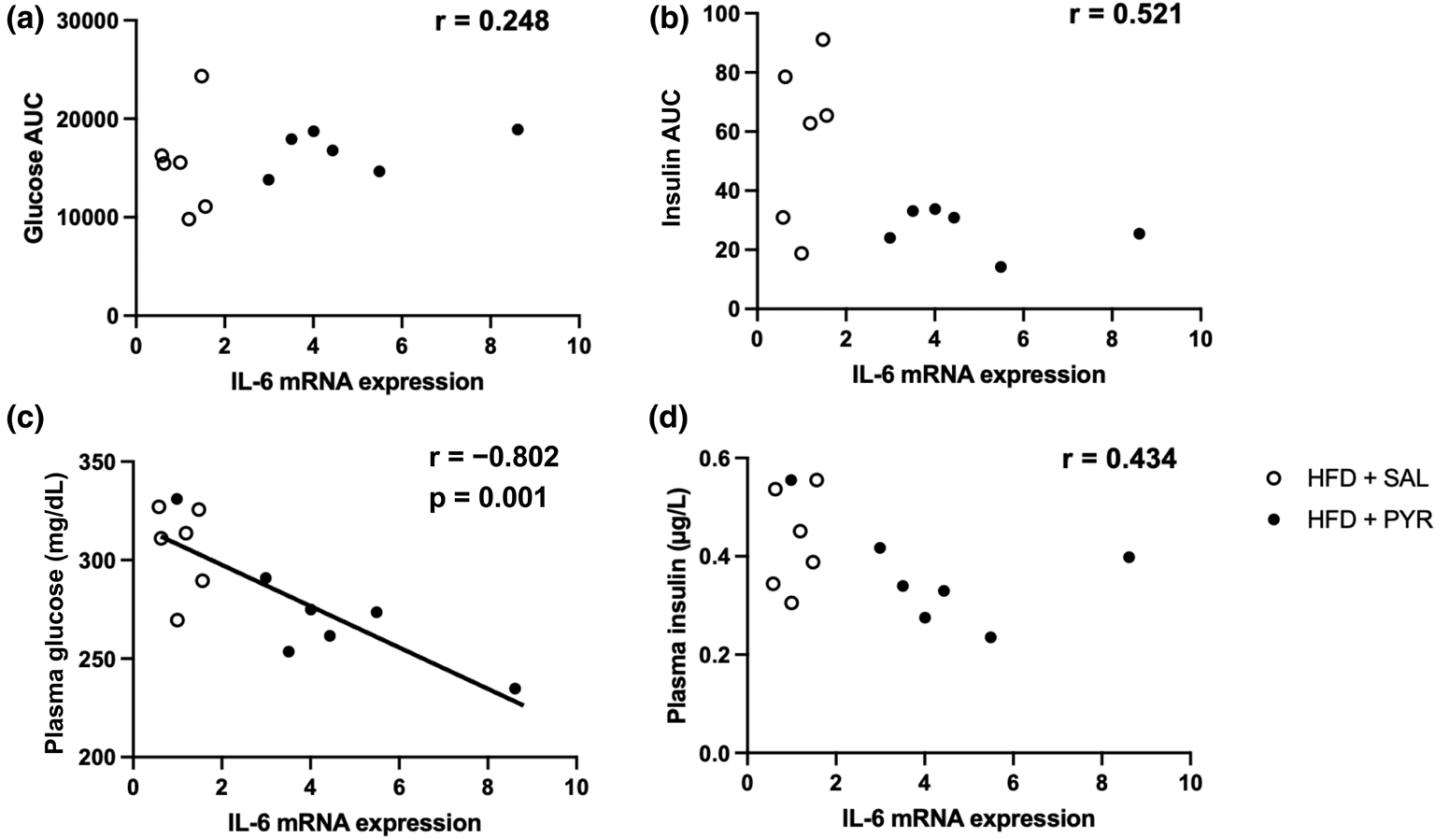
Relationship between interleukin 6 (IL‐6) mRNA expression levels and plasma glucose and insulin concentrations. Correlations between (a) IL‐6 mRNA expression and glucose area under the curve (AUC), (b) insulin AUC during an oral glucose tolerance test, (c) plasma glucose, and (d) plasma insulin concentrations in the basal state. *n* = 12–13.

Because IL‐6 has been shown to be related to whole‐body glucose metabolism (Carey et al., 2006; Peppler et al., 2019; Wan et al., 2012), correlation analysis between IL‐6 mRNA expression levels in eWAT and blood glucose and insulin concentrations was performed. To focus on the effects of pyruvate on HFD intake‐induced alterations, the analysis was performed in the HFD‐fed mice. There was no significant relationship between IL‐6 mRNA expression and the AUCs of glucose (Figure 6a) and insulin (Figure 6b) during the OGTT. IL‐6 mRNA expression showed a significant negative correlation with blood glucose (r = −0.802, *p* = 0.001; Figure 6c), but not insulin (Figure 6d) concentrations in the basal state.

### 3.8 Glucose metabolism‐related proteins in eWAT

**FIGURE 7:**
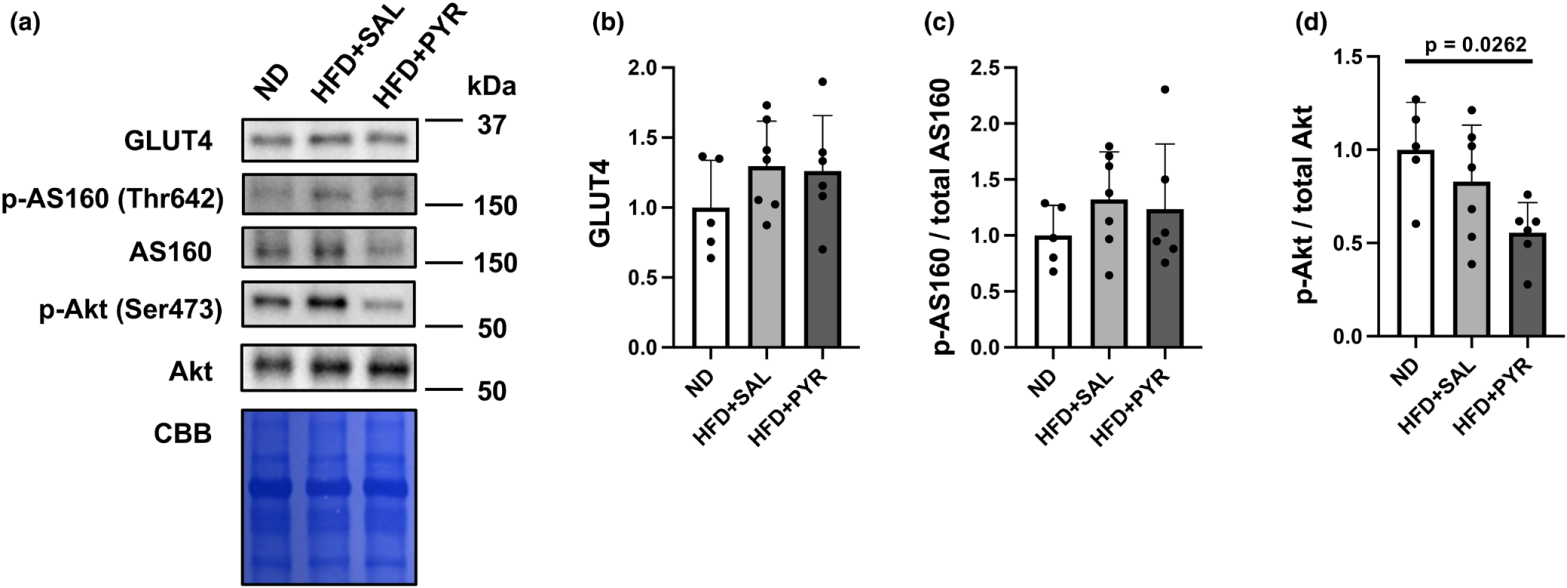
Glucose metabolism‐related proteins in epididymal white adipose tissue. (a) Representative images of Western blotting. (b) Glucose transporter 4 (GLUT4), (c) phosphorylated (p)‐Akt substrate of 160 kDa (AS160)/total AS160 ratio, and (d) p‐Akt/total Akt ratio. Data are shown as the mean ± standard deviation. ND: *N* = 5, HFD + SAL: *N* = 7, HFD + PYR: *N* = 6.

To investigate proteins related to glucose metabolism, glucose transporter type 4 (GLUT4) protein levels and the phosphorylation status of Akt substrate of 160 kDa (AS160) and Akt were measured in eWAT (Figure 7a). There were no significant differences in GLUT4 protein levels and AS160 phosphorylation status among the three groups (Figure 7b,c). Akt was phosphorylated significantly less in the HFD + PYR group compared to the ND group in the basal state (−44.5%, *p* = 0.0262; Figure 7d).

## 4 DISCUSSION

This study examined whether acute pyruvate administration increases lactate concentrations and whether chronic pyruvate administration alters the expression of inflammatory cytokines in adipose tissue and HFD‐induced systemic glucose metabolic dysfunction. The main results were as follows: (1) acute pyruvate i.p. injection increased lactate concentrations in eWAT and blood; (2) chronic pyruvate administration partially improved HFD‐induced systemic glucose metabolic abnormalities, that is, insulin concentrations at rest and during an OGTT; and (3) chronic pyruvate administration increased mRNA expression of inflammatory cytokines and a M2 macrophage marker and decreased mRNA expression of M1 macrophage and adipocyte differentiation markers in eWAT.

### 4.1 Effects of acute pyruvate administration on pyruvate and lactate concentrations in blood and tissues

Pyruvate i.p. injection significantly increased pyruvate and lactate concentrations in eWAT. This result indicates that, at least in adipocytes of eWAT, pyruvate is taken up and converted to lactate, resulting in lactate accumulation in eWAT. Adipose tissues have MCT1 protein, which has a high affinity for pyruvate (Bonen et al., 2006), with moderate mRNA expression in eWAT compared with muscle tissues (Lin et al., 2022). Although no studies have examined changes in lactate concentrations in adipose tissue after pyruvate administration, adipose tissue is recognized as an important site of lactate production in humans (Digirolamo et al., 1992; Hellström et al., 1997; Jansson et al., 1990) and rats (Newby et al., 1989; Thacker et al., 1987). Krycer et al. used a metabolomics approach to show that lactate production is prioritized in 3T3L1 adipocytes (Krycer et al., 2020). These previous studies and the present study support the hypothesis that lactate production is increased in adipocytes in eWAT after pyruvate administration. However, LDH activity is quite lower in adipose tissues than in skeletal muscles (Prochazka et al., 1970). Furthermore, because lactate uptake via MCT1 is competitively inhibited by pyruvate, it is possible that excessive pyruvate administration inhibited lactate uptake into peripheral tissues, leading to an increase in blood lactate levels. Therefore, although the contribution of eWAT to the increase in blood lactate concentration after pyruvate administration was unclear, at least pyruvate would be taken up into adipocytes and lactate was accumulated in the eWAT.

Moreover, the lactate/pyruvate ratio in eWAT was significantly increased after pyruvate administration. Since the cellular lactate/pyruvate ratio could be described as a proxy for the cytosolic NADH/NAD^+^ ratio (Mintun et al., 2004; Rabinowitz & Enerbäck, 2020), the increase in the lactate/pyruvate ratio suggests an increase in the NAD^+^/NADH ratio. A study using perfused rat hearts also revealed that the administration of 1 or 2 mM pyruvate decreases the NADH/NAD^+^ ratio, that is, it increases the NAD^+^/NADH ratio, in the myocardium (Park et al., 1998). This alteration of the NAD^+^/NADH ratio may be associated with an improvement in systemic glucose metabolism because it is well‐known that a reduction in the NAD^+^/NADH ratio causes metabolic abnormalities and age‐related diseases (Imai & Guarente, 2014). Taken together, the administered pyruvate would be converted to lactate in adipocytes in eWAT and possibly increased the NAD^+^/NADH ratio in this study.

### 4.2 Effects of chronic pyruvate administration on the HFD‐induced impairment of whole‐body glucose metabolism

Chronic pyruvate administration improved the AUC of the insulin concentration during the OGTT and resting insulin concentration in mice of the HFD‐PYR group, suggesting improved insulin sensitivity. These results are consistent with those of a previous study that used drinking water mixed with pyruvate to show that pyruvate administration prevents the deterioration of insulin sensitivity and systemic glucose metabolism (Hasan et al., 2024). Furthermore, chronic pyruvate administration increased the mRNA expression of inflammatory cytokines (i.e., TNF‐α, IL‐6, and MCP1) in eWAT, as we hypothesized. Interestingly, there was a significant negative correlation between IL‐6 mRNA expression and resting glucose concentrations in the two HFD‐treated groups. This result suggests that higher IL‐6 mRNA expression may be associated with the improvement of glucose metabolic dysfunction in HFD‐fed mice. It is reported that the AUCs of glucose and insulin concentrations during an OGTT are greater in IL‐6 knockout mice than in wild‐type mice after 10 weeks of HFD intake (Wan et al., 2012). Furthermore, acute IL‐6 administration improves whole‐body glucose metabolism in obese mice (Peppler et al., 2019). However, this relationship must be interpreted with caution because of several reasons. In this study, it was not known whether IL‐6 protein levels increased in the eWAT and blood after the intervention because we did not measure them. Furthermore, adipocyte‐specific IL‐6 deficiency did not markedly affect systemic insulin resistance in mice fed HFD (Han et al., 2020). Research using ob/ob mice lacking IL‐6 specifically in adipocytes concomitantly with a decrease in blood IL‐6 concentration revealed no significant alterations of glucose tolerance compared with normal ob/ob mice (Whitham et al., 2019). On the other hand, chronic infusion of IL‐6 for 5 days impaired whole‐body insulin sensitivity in mice (Klover et al., 2003). These results from previous studies suggest that increases in IL‐6 in adipocytes or blood do not necessarily improve glucose tolerance and insulin resistance. Alternatively, it is possible that the increase in blood lactate concentration due to pyruvate administration affected insulin sensitivity. It has been shown that lactate itself promotes browning white adipocytes (Carrière et al., 2014) and that visceral lactate concentration is related to insulin resistance (Feng et al., 2022). Therefore, there are several possibilities for the improvement of glucose metabolism by pyruvate administration, including effects of IL‐6 and/or lactate.

The improvement in resting insulin sensitivity following pyruvate administration in this study may be related to an increase in GLUT4 translocation rather than total GLUT4 protein content. This is because total GLUT4 protein levels were not significantly different between the HFD‐SAL and HFD‐PYR groups. AS160 phosphorylation status, which drives GLUT4 translocation to the plasma membrane in adipocytes (Sano et al., 2003), was comparable between both groups despite significantly lower resting insulin concentrations in the HFD‐PYR group than in the HFD‐SAL group. These data suggest that lower insulin concentrations were sufficient to translocate GLUT4 to the plasma membrane and maintain glucose concentrations in the HFD‐PYR group. A significant negative correlation between blood glucose concentration and IL‐6 mRNA expression in the eWAT was found in this study, but this intriguing relationship needs to be clarified further in the next study. Previous studies have examined the direct effects of IL‐6 using mice injected with IL‐6 neutralizing antibodies (Ikeda et al., 2016; Klover et al., 2005) and IL‐6 knockout mice (Han et al., 2020; Kurauti et al., 2017; Wan et al., 2012; Whitham et al., 2019) on glucose metabolism. Future studies using these mice should further clarify whether IL‐6 is involved in the improvement of insulin sensitivity and abnormal glucose metabolism induced by pyruvate administration.

### 4.3 Changes in the mRNA expression of several markers induced by chronic pyruvate administration

We consider the mechanism underlying the increase in the mRNA expression of inflammatory cytokines (i.e., TNF‐α, IL‐6, and MCP1) induced by chronic pyruvate administration to be the enhanced production and accumulation of lactate in adipocytes. Indeed, the expression of the inflammatory cytokine CD68 is increased in mice lacking MCT1 in adipocytes due to increased lactate accumulation in adipocytes because of their inability to release lactate (Lin et al., 2022). The adipocyte‐selective deletion of lactate dehydrogenase A, which converts lactate to pyruvate, is accompanied by a decreased percentage of inflammatory macrophages and the production of inflammatory cytokines such as IL‐1β (Feng et al., 2022). Along with increases in the expression of inflammatory cytokines observed in this study, NOS2, a marker of M1 macrophages, was decreased in the HFD + PYR group, while ARG1, a marker of M2 macrophages, was increased in the HFD + PYR group. In general, obese adipose tissue has a high number of M1 macrophages with insulin resistance, while healthy adipose tissue has a high number of M2 macrophages with higher insulin sensitivity (Rosen & Kajimura, 2024). These results for the expression of macrophage polarity markers suggest that adipose tissue in HFD + PYR mice was maintained in an insulin‐sensitive state. Moreover, the mRNA expression of PPARγ, C/EBPα, and C/EBPδ, which are markers of differentiation, was significantly decreased in the HFD + PYR group. This result is supported by another study showing a reduction in PPARγ mRNA expression and triglyceride accumulation with increased lactate release in 3T3L1 adipocytes with pharmacologically inhibited lactate dehydrogenase (Si et al., 2009). The changes in the mRNA expression of macrophage and differentiation markers are consistent with a previous study showing that pyruvate intake ameliorates HFD‐induced abnormalities in glucose metabolism (Hasan et al., 2024). However, in that study, inflammatory cytokine expression was rather suppressed, not increased, by chronic pyruvate intake. This discrepancy may be due to differences in the method of pyruvate administration; in this study, pyruvate was administered by i.p. injection, whereas in the study of Hasan et al., pyruvate was added to drinking water (Hasan et al., 2024). Therefore, the increase in the blood and tissue concentrations of pyruvate would be more transient and higher in this study than in their study, whereas in the previous study (Hasan et al., 2024), the increase in pyruvate concentration would be more sustained and lower than in our study. These differences in the pyruvate administration method may affect lactate concentrations and inflammatory cytokine mRNA expression in adipose tissue. It is necessary to clarify the effects of different types of change (sustained or transient) in pyruvate concentrations on the findings observed in this study.

### 4.4 Limitations

One of the limitations of this study is that only male mice were used. Increases in cytokine levels in adipose tissue (Vasconcels et al., 2019) and liver (Ortenzi et al., 2024) due to HFD intake have been shown to differ between male and female rats. In addition, female rats have enhanced cytokine production in response to pharmacological stimulation compared with male rats fed an HFD (Tibaes et al., 2022). The different effects of pyruvate administration on cytokine expression between male and female rats are unknown, but they should be clarified in future studies. Another limitation is that we assessed only eWAT after chronic pyruvate administration. The effects of pyruvate differ between cell types (Rao et al., 2021). For example, pyruvate upregulates hepatic fibroblast growth factor 21, which regulates metabolic function (Zhao et al., 2022), and activates mitochondrial biogenesis in skeletal muscle‐derived C2C12 cultured cells (Philp et al., 2010; Wilson et al., 2007). It is also suggested that the improvement of glucose metabolism due to IL‐6 may be mediated by the liver (Peppler et al., 2019). Therefore, it should be kept in mind that the improvement of whole‐body glucose metabolism by pyruvate administration may be associated with alterations of other tissues that were not examined in this study.

## 5 CONCLUSION

The aim of this study was to clarify whether pyruvate administration changes inflammatory cytokine mRNA expression in adipose tissue and to determine whether it improves the HFD‐induced dysfunction of whole‐body glucose metabolism in mice. We found that pyruvate administration transiently increased lactate accumulation in blood and eWAT. Chronic pyruvate administration partially improved systemic glucose metabolism and inhibited an increase in adipose tissue weight in HFD‐fed mice. The intervention also altered several mRNA expressions in the eWAT such as increased inflammatory cytokines and an M2 macrophage marker and decreased adipocyte differentiation and a M1 macrophage markers. This study provides the possibility that pyruvate administration is an intervention to improve HFD‐induced glucose metabolism dysfunction, but the molecular mechanism potentially including increases in IL‐6 and blood lactate concentration needs to be further elucidated.

## AUTHOR CONTRIBUTIONS

TA and DH conceived and designed the research, prepared the figures, and drafted the manuscript. TA, RT, RK, TK, and DH performed the experiments. TA, RT, RK, TK, YN, YK, and DH analyzed and interpreted the data, edited and revised the manuscript, and approved the final version of the manuscript.

## FUNDING INFORMATION

This study was supported by the grants from Japan Society for the Promotion of Science (20H04071 and 20K02812).

## CONFLICT OF INTEREST STATEMENT

The authors declare no conflict of interest.

## ETHICS STATEMENT

This study was approved by the Institutional Animal Care and Use Committee of the University of Electro‐Communications.

## Data Availability

Data will be made available upon request to the corresponding author.
